# The Exploration of Complement-Resistance Mechanisms of Pathogenic Gram-Negative Bacteria to Support the Development of Novel Therapeutics

**DOI:** 10.3390/pathogens11080931

**Published:** 2022-08-18

**Authors:** Marta K. Ruest, Jonathan J. Dennis

**Affiliations:** Department of Biological Sciences, University of Alberta, Edmonton, AB T6G 2R3, Canada

**Keywords:** complement system, bacteria, innate immunity, gram-negative bacteria, complement resistance

## Abstract

Resistance to antibiotics in Bacteria is one of the biggest threats to human health. After decades of attempting to isolate or design antibiotics with novel mechanisms of action against bacterial pathogens, few approaches have been successful. Antibacterial drug discovery is now moving towards targeting bacterial virulence factors, especially immune evasion factors. Gram-negative bacteria present some of the most significant challenges in terms of antibiotic resistance. However, they are also able to be eliminated by the component of the innate immune system known as the complement system. In response, Gram-negative bacteria have evolved a variety of mechanisms by which they are able to evade complement and cause infection. Complement resistance mechanisms present some of the best novel therapeutic targets for defending against highly antibiotic-resistant pathogenic bacterial infections.

## 1. Introduction

Antibiotic resistance as produced by bacterial cells has existed long before human beings began using antibiotics as chemotherapy to treat bacterial infections [1,2,3,4]. However, due to the frequent use and misuse of antibiotics, antibiotic resistance is now on track to become the major causative mechanism behind the next global pandemic. This phenomenon of widespread antibiotic resistance is a consequence of the ability of bacterial cells to share genetic information through horizontal gene transfer (HGT) [1,5]. As a result, many commensal and pathogenic bacteria now possess the molecular machinery to resist multiple chemical structural classes of antibiotics [1,6,7]. Although antibiotic-resistant bacterial infections may be difficult to treat with current therapeutics, often requiring the long-term application of several complementary frontline antibiotics, the real issue at hand is the difficulty in developing novel antibiotics for which resistance does not yet exist. After over 20 years of modern antibiotic discovery efforts, few new drugs with novel or effective mechanisms of action have been developed and brought to market [1,8,9]. This is a serious issue considering that 2.8 million individuals worldwide are infected with antibiotic-resistant bacteria per year, of which, on average, 35,000 people will die [10]. People who are immuno-compromised are the most at risk of acquiring multi-drug resistant (MDR) infections and are typically infected with highly antibiotic-resistant opportunistic Gram-negative bacterial pathogens [1,6,11].

Gram-negative bacteria present some of the greatest threats to human life in terms of antibiotic resistance. There has been a significant increase in Gram-negative bacterial infections worldwide in the past several decades, and the majority of hospital-acquired or nosocomial infections are now caused by Gram-negative bacteria [12,13]. Additionally, the US Center for Disease Control (CDC) has declared MDR Gram-negative pathogens as the most substantial threat to humanity during this antibiotic resistance era [10]. Gram-negative bacteria not only possess intrinsic antibiotic resistance mechanisms but are also adept at acquiring various extrinsic antibiotic resistance mechanisms through HGT [1,2,6,14,15].

It is suspected that the unique structure of the Gram-negative cell envelope is what confers high levels of antibiotic resistance to these bacteria [1,6]. Together, the inner phospholipid bilayer membrane, the periplasm, the thin layer of cross-linked peptidoglycan, and the asymmetrical outer membrane significantly decreases membrane permeability, rendering Gram-negative bacteria intrinsically resistant to multiple classes of antibiotics [1,6]. The tight packing of lipid A molecules in the outer membrane is also in part responsible for the impenetrability of these cells [1,6,14,15,16]. Additionally, Gram-negative bacteria use a variety of efflux pumps spanning portions of, or the entire cell envelope, to remove waste materials from the cytoplasm and periplasm, which are also capable of pumping out antibiotics [1,2,6]. The combination of a relatively impermeable cell envelope structure, plus a variety of efflux pumps actively extruding a wide range of chemical compounds, makes it particularly difficult to develop novel antibacterial compounds effective against Gram-negative bacterial pathogens [1,6]. These unique intrinsic features impose a wide range of chemical restrictions on effective antibiotic compounds and have resulted in the need for extensive small molecule screening in an effort to discover novel antibiotic candidates [1,17]. Some of these screens have been largely ineffective because they are typically conducted under artificial conditions where many of the bacterial processes that are essential for causing an infection are not expressed [18]. Consequently, effective drug screens for potential candidate compounds require both in vitro identification and in vivo efficacy validation.

For a bacterium to cause infection in a human host, it must elaborate on a number of defense and virulence factors. These can range from tissue degradation enzymes and cellular toxins to immune evasion molecules. In the field of antibiotic drug discovery, bacterial virulence factors are becoming important potential target molecules. Antibiotics targeted against bacterial virulence factors will not directly kill the bacterium; however, they will render the bacterium more susceptible to complementary host immune responses [19,20]. Because these anti-virulence antibiotics will not directly kill the bacterium, they will decrease selection pressure on the bacterial population to mutate towards resistance and, in turn, reduce the chance of antibiotic resistance developing [19,20]. Any bacteria that develop resistance through antibiotic target alteration will more likely destroy the functionality of the virulence factor, thus rendering these variants more susceptible to the host immune system. This “anti-virulence strategy” has led to the idea that some of the best therapeutic targets are bacterial immune evasion molecules, and those targets which allow the bacterium to avoid the innate immune system have been of particular interest. The complement system is part of the innate humoral immune response, and it is one of higher organisms’ first lines of defense against bacterial pathogens. Any pathogen entering the body must be able to evade this defensive immune system, and mechanisms used by pathogenic bacteria to evade complement present some of the best novel therapeutic targets.

The following article will describe the human complement system’s response to invading Gram-negative bacterial pathogens. Subsequently, complement system regulation will be discussed, which ensures only pathogenic organisms and not host cells are attacked. Finally, bacterial complement evasion strategies used by various Gram-negative bacteria will be described, and these mechanisms may serve as promising therapeutic target candidates.

## 2. The Complement System

The complement system is a component of the innate, humoral immune system that plays a variety of roles in maintaining homeostasis [21]. Complement has been identified as playing major roles in typical immune development, certain autoimmune diseases, coagulation responses, and various common illnesses such as arthritis [22]. The complement system is the first line of bodily defense against pathogenic invaders, including viruses, bacteria, fungi, and parasites, including protozoa, helminths, and ectoparasites [23,24,25]. The complement system achieves this by bridging the gap between innate and adaptive immunity playing a role in B-cell differentiation and maintenance, as well as T-cell activation [26]. The complement system, also termed the complement cascade, comprises 60 different effectors, mostly proteins, that are directed toward tagging and destroying pathogens [27]. However, the complement system by itself is only capable of directly killing certain classes of pathogens, including Gram-negative bacteria. Due to their outer membrane and a thin layer of peptidoglycan, Gram-negative bacteria are the major class of pathogens predominantly susceptible to lysis by the process of complement-mediated killing, which is detailed in the following paragraphs [25].

**Figure 1:**
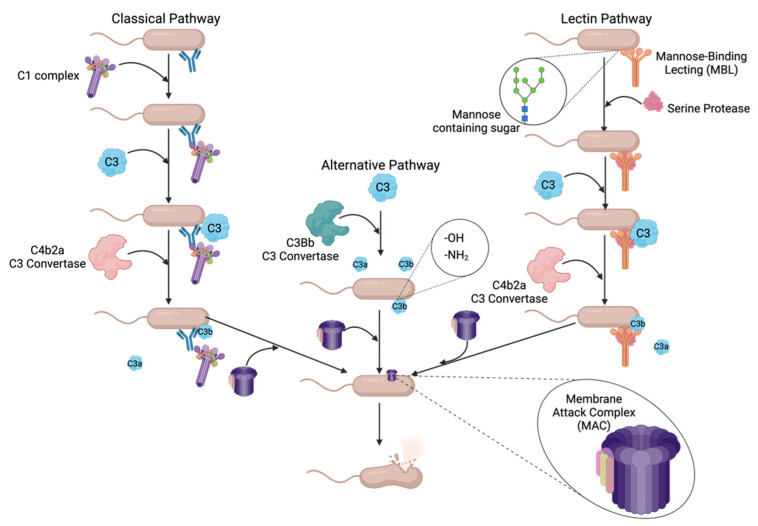
Overview of complement cascade in relation to cell lysis of Gram-negative bacteria. Complement uses three different pathways to recognize bacterial pathogens: classical, alternative and lectin. The classical pathway uses antibodies to recognize specific antigens on a bacterium. The alternative pathway is constitutively active and deposits C3b molecules on bacterial surfaces with -OH or NH_2_ groups exposed. Lastly, the lectin pathway will recognize specific sugar structures on the outer surface of the bacteria. Once the bacterium has been identified and tagged by the initial components of the respective complement pathway, they all converge at the deposition of C3b on the outer membrane. Once C3b has opsonized a pathogen MAC assembly will begin leading to the insertion of a pore in the outer membrane which results in cell lysis. Created with BioRender.

Complement can recognize a pathogen using one of three pathways, the alternative, classical, and/or lectin pathways, that each leads to a common terminal cascade. Figure 1 outlines how these first three pathways identify and tag a pathogen for killing by the terminal pathway, which in turn make up the complement cascade. The alternative, or surveillance complement pathway, is constitutively active and does not identify or recognize any specific bacterial surface structure; instead, active C3b protein binds to -OH or -NH2 groups on an amino acid of bacterial surface proteins [21]. The classical complement pathway uses C1q protein to initiate the cascade by binding to either lipopolysaccharide (LPS) found only in Gram-negative bacteria, bacterial outer membrane proteins, or an antibody bound to a bacterium [21,23]. C1q will then recruit serine proteases C1s and C1r to the bacterial surface, which will, in turn, recruit C3 and a C3 convertase [25]. Lastly, the lectin pathway uses mannose-binding lectin (MBL) to recognize specific molecular patterns on the surfaces of pathogens leading to the formation of the MBL-associated serine protease (MASP) complexes [25,26]. MASP complex will then recruit C3 and a C3 convertase as in the classical pathway [25].

Once a pathogen has been recognized by one of the three pathways, the complement cascade converges at the cleavage of C3 protein into its active constituents, C3a and C3b, using a C3 convertase [21]. The alternative pathway uses the C3bBb convertase generated from either the Factor B or Factor D serine proteases [21]. The classical and lectin pathways utilize the C4b2a C3 convertase generated from the cleavage products of C4 and C2, recruited by each of the pathways’ respective serine proteases [21,25]. Once the active C3b cleavage product is generated and deposited onto the bacterial surface, the terminal pathway will be initiated.

After a Gram-negative bacterium has been opsonized by multiple C3b proteins, the C3 convertases exhibit decreased specificity and begin to cleave C5 protein into C5a and C5b [21,25]. C5b then serves as a molecular scaffold for the construction of the membrane attack complex (MAC) [28]. C5b is an unstable protein until bound by protein C6 creating the stable, soluble C5b-6 complex [28]. Next, C7 protein is added, rendering the complex lipophilic, which is required for its insertion into the amphipathic outer membrane [28]. C8 is added to form the C5b-8 complex, which is inserted into the bacterial outer membrane [28]. Lastly, multiple copies of the C9 protein are added to form a ~10 nm pore in the outer membrane resulting in cell lysis [23,28].

Although insertion of the MAC results in cell death, the molecular mechanism by which cell death occurs has yet to be experimentally confirmed. The most accepted theories suggest that insertion of MAC into the outer membrane causes a decrease in membrane potential and a large influx of water which results in lysis [23]. Many experts argue that insertion of the MAC into the outer membrane alone would not be sufficient to cause cell lysis and suggest that the MAC actually spans across the outer membrane, peptidoglycan layer, and the inner membrane. Others argue that the initial insertion of the MAC into the outer membrane allows for further MACs to assemble in the inner membrane [23]. Alternatively, Kashyap et al. [29] suggest that insertion of the MAC into the outer membrane is detected by the bacterial cell and causes initiation of an apoptosis-like response leading to cell destruction.

Although the main objective of the complement system is to defend against pathogenic invaders, the response may also indirectly result in host cell damage. This damage is caused by the generation of anaphylatoxins C3a and C5a from the cleavage of C3 and C5, respectively. When present in high enough concentrations, these molecules initiate an inflammatory response that leads to the generation of free radicals that damage cells non-specifically [25,30]. Bloodstream infections are particularly dangerous, as the complement system mounts a large response towards the bacterial invader, thus generating large amounts of C3a and C5a [30]. When present in the blood, these inflammatory molecules will disseminate rapidly throughout the body and can cause systemic organ failure [30]. However, the production of C3a has also been demonstrated to have additional antibacterial effects [23,31]. It was observed that C3a was structurally homologous to other known antimicrobial peptides, and subsequent experiments concluded that C3a was able to kill both Gram-positive and Gram-negative bacteria [31]. It is likely that the generation of C3a in small to moderate amounts would be beneficial in helping the host protect itself from bacterial pathogens.

## 3. Complement Regulation

**Figure 2:**
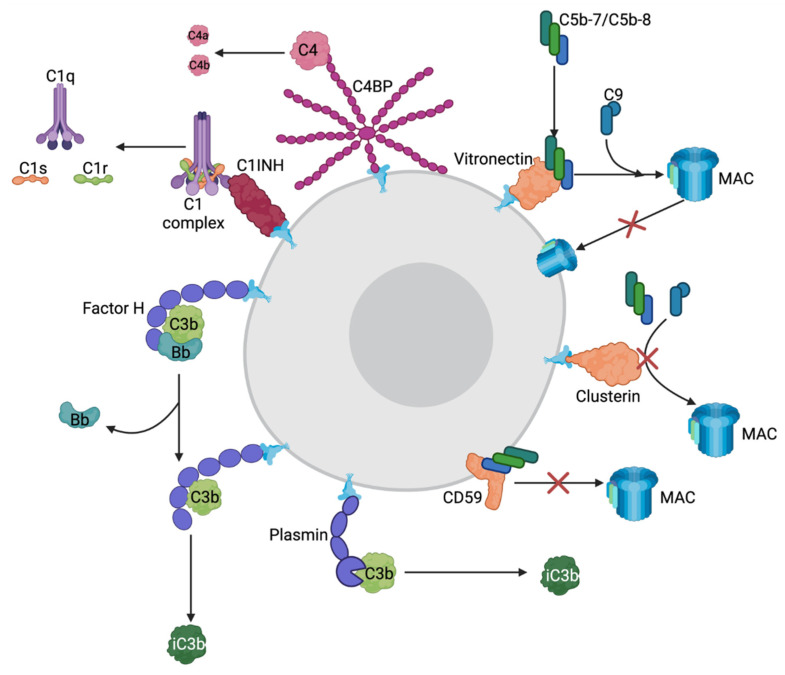
Mechanisms of host-produced complement-regulator molecules. Purple molecules = alternative pathway regulators; magenta = classical/lectin pathway regulators; orange = terminal pathway regulators. Factor H can bind C3 convertase C3bBb causing Factor Bb to dissociate rendering the convertase inactive. Factor H can also bind C3b and act as a cofactor to Factor I cleaving it into inactive C3b. Plasmin, the active zymogen of plasminogen, can also bind C3b and act as a cofactor to Factor I cleaving it into inactive C3b. C4BP can bind C4 and cleave it into C4a and C4b using Factor I. C1INH binds C1 complexes and dissociates them into C1, C1s and C1r. Vitronectin binds C5b-7/C5b-8 and allows full assembly of the MAC but prevents it from being inserted into the membrane. Clusterin binds C5b-7/C5b-8 and stops it from association with C8 and C9 preventing MAC assembly. CD59 binds C5b-8 and prevents MAC assembly. All these mechanisms are used by human host cells to prevent complement-activation but bacteria are also able to acquire these proteins to their cell surface protecting them against complement. Created with BioRender.

Because complement can cause life-threatening reactions, the system must be carefully monitored and regulated to keep the cascade directed towards the proper cells. This control requires numerous regulatory proteins to differentiate healthy host cells from invading pathogens [32]. There are three general classes of complement-regulator molecules: fluid phase, host surface attached, and integral membrane complement clearance receptors, which are mostly proteins [32]. Surface attached and integral membrane regulators protect against attack from all three complement pathways and are, for the most part, unable to be recruited by bacteria to protect themselves against complement [32]. In contrast, individual fluid phase regulators are specific and provide protection against one or two of the complement pathways and are easily recruited to the outer membrane of Gram-negative bacterial pathogens to disguise themselves as host cells [32]. For these reasons, this review will focus mostly on fluid phase complement regulators, as summarized in Figure 2 and Table 1.

The most abundant fluid phase regulator is Factor H (FH), which negatively regulates the alternative pathway [32]. FH dismantles the complement response by inactivating or preventing further activation of C3b in many ways, ultimately preventing the deposition of C3b on a cell’s surface. To prevent further activation and amplification of the complement cascade, FH prevents C3b from interacting with Factor B to generate C3bBb, the alternative pathway C3 convertase [33]. Additionally, if a C3b molecule is attached to the bacterial cell surface in proximity to an FH molecule, FH will act as a cofactor to the serine protease Factor I, which will modify C3b into inactive C3b [21,32]. Lastly, FH will decrease alternative pathway activation by dissociating the C3bBb convertase into its constituents, C3b and Bb, upon binding [32].

Additionally, the FH gene is capable of creating many variants of the protein due to alternative splicing/differential gene expression [34]. These include FH-like (FHL) proteins which result from alternative splicing of the FH gene and are negative regulators of complement [35]. FHL-1 is a truncated version of FH and also acts as a cofactor to Factor I to cleave C3b into inactive C3b [32]. Although FH and FHL-1 perform the same function, they perform it in different regions of the body [34]. More specifically, in areas where the protein needs to cross membranes to get to its final destination, the truncated FHL-1 is more favorable than the full-length FH [34]. There are also FH-related (FHR) proteins that share sequence homology to FH but do not contain the canonical complement regulatory domains [35]. FHR-1 is characterized as inhibiting the C3 convertase of the alternative pathway, thus preventing it from converting C3 into its active constituents [32,36]. Additionally, FHR-1 can also bind C5 and C5b-6 to stop further progression of the terminal pathway. Interestingly, FHR-1 has also been demonstrated to activate the alternative pathway by binding C3b and providing a scaffold for C3bBb construction [35]. Research into this discrepancy is limited, so we hypothesize that FHR-1 can potentially exist in two different states that determine whether the protein activates or inhibits complement.

Plasminogen (PLG) is another fluid phase regulator of complement with multiple physiological roles as an enzyme that is capable of breaking down various tissue barriers and preventing blood clots from becoming too large via fibrinolysis [37]. In the complement system, PLG mainly regulates the classical and lectin pathways; however, it also plays a minor role in regulating the alternative pathway [38]. PLG can be converted into its active form, plasmin, by various proteases originating from the host or from invading bacteria. Plasmin is subsequently capable of binding either C3 or C5 and cleaving them into inactive forms using its serine protease domain [38]. Plasmin can also bind C3b, whereby it will increase the rate of cleavage by Factor I resulting in inactive C3b regulating the alternative pathway. Because PLG plays a role in both fibrinolysis, degradation of blood clots, and complement systems, it is highly favorable for bacterial pathogens to acquire this protein to its surface as a means of immune evasion via cloaking, and thus, PLG binding proteins have been identified in the majority of bacterial pathogens [38].

C4 binding protein (C4BP) is one of the major regulators of the complement system controlling the classical and lectin pathways [32,39]. C4BP binds C4b and acts as a cofactor to Factor I resulting in cleavage and inactivation of C4b. Additionally, C4BP blocks the formation of the C3 convertase C4b2a of the classical and lectin pathways by binding C4b and accelerating its decay. Lastly, C4BP can assist in the degradation of C3b to inactive C3b as a cofactor to Factor I, thereby regulating the alternative pathway to a minimal extent [32,39].

C1 inhibitor (C1INH) is a negative fluid phase complement regulator of the classical and lectin pathways [32]. C1INH binds C1s and C1r dissociating them from C1q preventing further progression of the complement cascade [32,40]. C1INH can also bind MASP-1 and MASP-2 of the lectin pathway, preventing them from associating with their respective bacterial receptors [32,40]. CINH is also capable of preventing the formation of the alternative pathway C3 convertase by preventing the association of C3b and Factor B/Factor Bb [40].

Although all these mechanisms exist to regulate the initiation of the complement cascade, the complement pathway can still be accidentally activated against host cells; thus, terminal pathway regulators are also required. There are two main fluid phase regulators of the terminal pathway: vitronectin (Vn) and clusterin (Cn) [32]. Vn binds the C5b-7 terminal complex and allows the MAC to fully assemble using C8 and multiple copies of C9; however, it prevents insertion of the MAC into the cell membrane [32,41,42,43,44]. Vn may also bind C5b-8 complexes and prevent the association with C9 to stop the formation of the MAC [28,32]. Cn performs a very similar function to Vn in that it binds to soluble C5b-7 and C5b-8 prior to entering the outer membrane [28,32]. Unlike Vn, Cn can also bind soluble C5b-9 and inhibit further formation or insertion of the MAC [28,42,44]. Lastly, there is also an integral membrane regulator of the terminal pathway CD59, also known as MAC inhibitory protein or protection [28,32]. CD59 is the main negative regulator of the terminal pathway because it prevents the association of C9 with the rest of the MAC by binding membrane inserted C5b-8 complexes [28,32]. CD59 is commonly shed from host membranes and, in some cases, can be recruited into bacterial outer membranes for complement evasion [32].

Most complement regulators inactivate complement molecules. However, there are some complement regulators that are known to activate complement. Properdin is a positive complement regulator of the alternative pathway that functions by stabilizing the C3 convertase C3bBb to increase its half-life [32,41]. Properdin interacts with C3b and Factor B and acts as a molecular scaffold for their association to form the active convertase, C3bBb [45]. Because the alternative pathway is constitutively active, this mechanism likely exists to save host resources and energy by producing fewer complement components. It is interesting that the classical and lectin pathways do not have positive regulators, but possibly a positive regulator here would cause dangerously high levels of complement activation that would lead to the production of large amounts of anaphylatoxins C3a and C5a.

Interestingly, some pathogens purposefully activate the complement system in order to assist in the causation of disease, which is sometimes known as the “Hitchhiking principle” [46]. This principle is used by intracellular pathogens such as *Mycobacterium tuberculosis* and many viruses whereby they recruit a complement protein to their membrane or surface in order to enter host cells [47,48]. Various other pathogens also bind FHR-1, and conflicting evidence suggests that bound FHR-1 can both activate and inhibit the complement system, as discussed earlier [35]. FHR-1 bound to *Staphylococcus aureus* demonstrated no protective effect against complement mediated-killing and sometimes even increased complement activation on the pathogen’s surface [35]. Therefore, the molecular context of FHR-1 binding possibly plays a role in determining whether FHR-1 will increase or decrease complement activation. Regardless, pathogens that recruit complement-activating proteins must possess many complement-resistance mechanisms to handle the extra stress that comes with increased complement surveillance. Lambris et al. [47] hypothesized that because many non-intracellular bacterial pathogens possess a large and diverse arsenal of complement-resistance mechanisms, they also may be activating the complement response to increase their ability to cause infection.

## 4. Complement-Resistance Mechanisms and Application to Therapeutics

The most common complement-resistance mechanism utilized by invading bacteria is the acquisition of host-produced complement-regulator molecules, either through their addition to a bacterial cell surface protein or, in rare cases, by incorporating them into the bacterial outer membrane. Because different pathogenic bacteria are not recognized by all pathways of complement, in those cases, it is not necessary to express complement-evasion mechanisms against all pathways. Table 2 lists some of the more well-characterized complement resistance mechanisms discovered in Gram-negative bacteria.

### 4.1. Classical Pathway Resistance

Lathem et al. [46] discovered that the secreted StcE metalloprotease of *Escherichia coli* is capable of binding C1INH. It was also found that StcE is capable of cleaving C1INH, which likely relates to its ability to inhibit the contact activation pathway, which is involved in coagulation and leads to the formation of thrombin or blood clots that prevent any further spreading of bacterial infections [49]. C1INH is the only protein known to be involved in the regulation of both the complement and contact activation pathways [49]. Therefore, StcE plays a dual role in virulence rendering it a good potential antibiotic target. Marr et al. [50] determined that the *Bordetella pertussis* surface protein Vag8 binds C1INH as well, protecting *B. pertussis* from complement-mediated lysis. Unfortunately, these authors did not investigate whether this interaction plays a role in contact activation. It was concluded that Vag8 expression was controlled by the previously identified Bvg two-component system. This two-component system has been thoroughly studied and is understood to regulate the expression of a wide variety of virulence factors, but it also specifically controls many complement-resistance genes. It could be of potential interest to create a drug capable of targeting and inhibiting this two-component system by preventing it from turning on the expression of these virulence-related genes. Bikard et al. [51] researched the potential of designing sequence-specific CRISPR-Cas-based antibacterial agents, which may be a strategy used to inhibit the Bvg two-component system. The researchers used a phage delivery system and found it to be effective in killing bacteria by inducing double-stranded breaks in the genome. The authors also discuss that these therapeutics could potentially target multiple virulence factors using the ability of the CRISPR-Cas system to act on many targets. A CRISPR-Cas antibacterial directed at the Bvg two-component system could directly kill the bacteria and decrease the likelihood of resistance developing because multiple virulence factors are being targeted.

*B. pertussis* and *E. coli* both have proteins that also bind C4BP to protect themselves from the classical and lectin pathways of complement. *B. pertussis* uses the Filamentous Hemagglutinin (FHA) protein to bind C4BP [52]. FHA was originally characterized as being an adhesion structure and capable of causing autoagglutination [53,54]. It was also identified that FHA could either be surface-attached or secreted, making it an interesting therapeutic target [54]. *E. coli* uses the outer membrane protein OmpA to bind C4BP, as discovered by Prasadarao et al. [55]. They designed a synthetic peptide that would bind OmpA preventing it from interacting with C4BP and found that this significantly reduced the bacterium’s ability to survive complement-mediated killing. This is proof of the concept that inhibiting complement-resistance factors does render bacteria sensitive to complement-mediated killing and that these targets have the potential to be of use in antibiotic discovery.

*E. coli* also possesses a unique classical pathway complement-resistance mechanism that has yet to be discovered in any other species to date. Biesecker et al. [56] identified that curli proteins increase the amount of C1q on the bacterial surface; however, they have no effect on C3b deposition. The authors concluded that curli proteins must be inhibiting one or many steps of the complement cascade between C1q binding and C3b deposition. For this to be useful in antibiotic development, the exact molecular mechanism by which curli is inhibiting the further progression of the complement cascade must be characterized. Additionally, curli is also used by *E. coli* cells to establish a community and form biofilms when causing infection [57]. Due to its multiple roles in virulence, curli is of particular interest for antibiotic development, as inactivation of these proteins would likely render them non-functional in at least one of their virulence-related functions, making these treated *E. coli* cells less virulent.

Liu et al. [58] showed that when *E. coli* loses expression of outer membrane protein OmpC, it renders the cells resistant to the classical pathway of complement. Under normal circumstances, OmpC recruits C1q to the bacterial surface, thereby initiating the complement response against the bacterium. Loss of the OmpC protein then decreases C1q deposition and thus the overall complement response. The authors note that since some antibiotics require a functional OmpC in the outer membrane to cross into the cell envelope and interact with their target molecule, the loss of expression of OmpC imparts increased antibiotic resistance for these cells. Interestingly OmpC has also been implicated in playing a role in the Mla pathway, the pathway responsible for maintaining lipid asymmetry with LPS in the outer membrane, which is crucial for immune evasion of Gram-negative bacteria [59,60,61]. In terms of therapeutics, it may be of interest to maintain OmpC expression so that complement remains active in recognizing the bacterial surface and so antibiotics that require OmpC, such as carbapenems and cephalosporins, can still enter the cell.

Although classical and lectin pathway resistance is of interest, these pathways must be turned on by different immune effectors to perform their function. In contrast, the alternative complement pathway is constitutively active; thus, pathogens must be prepared to defend themselves against this pathway immediately upon entry of the host [25]. Therefore, resistance to the alternative complement pathway is critical for many pathogens.

### 4.2. Alternative Pathway Resistance

Factor H is the main negative regulator of the alternative pathway. This has resulted in a wide variety of bacterial species expressing surface proteins that are able to recruit the protective FH to the outer membrane. For example, the Gram-negative bacterium *Acinetobacter baumannii* presents one of the largest threats to humans in terms of antibiotic-resistance infections, as some clinical isolates have been found to be pan-resistant to all clinically available antibiotics [1]. For this reason, some experts in the field have deemed *A. baumannii* untreatable [1]. Kim et al. [62] identified that *A. baumannii* uses OmpA to bind FH and, in turn, resist complement-mediated killing. It is also known that *A. baumannii* OmpA plays a role in a diverse set of cellular processes, including invasion and induction of apoptosis in host cells and bacterial adhesion to host cells [63]. Again, bacterial surface proteins such as OmpA with multiple roles in virulence are of particular interest for novel therapeutic targets due to the decreased likelihood of resistance development. *N. meningitidis* also uses a surface-exposed protein to bind FH, called NspA [64]. It was found that the molecular context of this interaction affected how well FH could bind to NspA. In particular, sialylated lipooligosaccharide increased the ability of NspA to bind FH [64]. Estabrook et al. [65] also determined that sialylated lipooligosaccharide was in part responsible for serum resistance in *Neisseria* sp. Thus, both sialylated lipooligosaccharide and NspA contribute to the binding of FH and the subsequent inhibition of the alternative complement pathway.

Although binding FH alone is an effective complement-resistance strategy, many pathogens spread throughout the body to regions where FH is not present. Consequently, they bind alternatives to FH, such as FHL or FHR proteins. *P. aeruginosa* possesses the Lpd surface-exposed protein that is capable of binding FH, FHL-1, FHR-1, and PLG to protect itself from the alternative and terminal pathways of complement [66]. As previously mentioned, PLG can also be used as a tissue degradation factor [37]. The binding of PLG to the bacterial membrane plays dual roles in complement protection and infection spreading. Because *P. aeruginosa* can cause system-wide infections, it must be able to bind the truncated version of FH, FHL-1, in order to be protected from the alternative pathway in all areas of the body [34]. Furthermore, due to the fact that the alternative pathway is always active, *P. aeruginosa* also uses surface-exposed EF-Tuf to bind FH, FHL-1, and PLG for complete protection [67]. *A. baumannii* also uses EF-Tuf localized to the outer membrane to bind PLG [68]. EF-Tuf is canonically used as a translation elongation factor in the cytoplasm but can be exported to the outer membrane to play multiple roles in virulence [69]. Proteins that exhibit multiple functions are termed “moonlighting proteins”.

**Figure 3:**
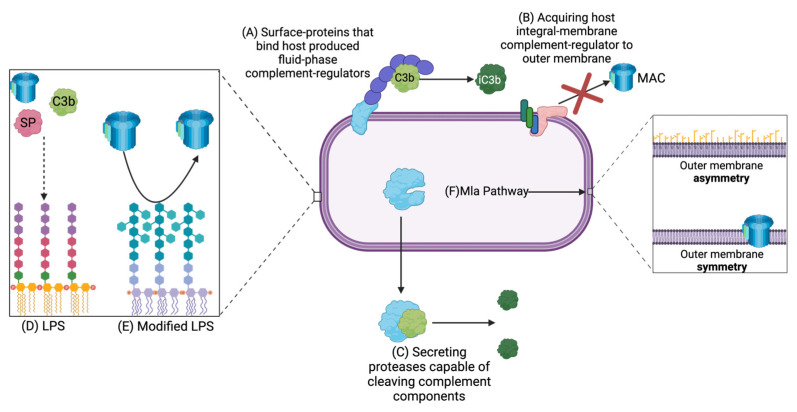
Bacterial mechanisms used to evade complement-mediated killing. (**A**) Production of surface-exposed proteins that can bind host-produced fluid-phase complement-regulators which typically inactivate specific complement components. (**B**) Acquiring host integral-membrane complement-regulator CD59 to prevent MAC insertion into outer membrane. (**C**) Secretion of proteases that can cleave complement components into inactive forms. (**D**) LPS causes steric hindrance making it more difficult for complement components to bind or be inserted to/into outer membrane, some insertion still occurs with traditional LPS however. (**E**) LPS modified with additional sugars increases steric hindrance and prevents MAC insertion. (**F**) Mla pathway is responsible for maintaining lipid asymmetry in the outer membrane, i.e., LPS can cause steric hindrance preventing MAC insertion, when Mla pathway is non-functional due to mutation or blockage in the pathway the outer membrane is composed of phospholipids only and MAC can be inserted. Created with BioRender.

Moonlighting proteins were discovered by Piatigorsky and Wistow in 1989 and are defined as being proteins that are capable of performing two separate physiological functions while exhibiting the same overall 3D structure [70]. In bacteria, these proteins usually perform one essential biological process in the cytoplasm and typically play a role in virulence when translocated to the outer membrane [70,71]. Recent research into moonlighting protein structure differences within the cytoplasm and outer membrane revealed that covalent interactions differ slightly in the protein depending on its localization and may permit them to perform different functions [72]. Some experts suggest that the majority of moonlighting proteins are used for immune evasion when exported to the outer membrane and that the majority of them are capable of binding host-produced proteins [73,74,75]. Franco-Serrano et al. [71] hypothesized that bacteria use moonlighting proteins for virulence because they are so well-conserved between host and bacteria due to their roles in key ancestral biological processes. Therefore, protein homology between host and bacterial proteins decreases the likelihood of an immune response being elicited against the bacterium. A therapeutic agent that inefficiently crosses the cell envelope will force the drug to interact with the secreted form of the moonlighting protein, thus reducing bacterial virulence but also decreasing resistance selection (Figure 3). If the drug does cross the cell envelope to bind the cytoplasmic moonlighting protein, however, it will also directly kill the pathogen by inhibiting its target essential cytoplasmic function.

### 4.3. Terminal Pathway Resistance

Although it may seem beneficial to stop the complement cascade in earlier steps, many bacteria also possess terminal pathway resistance mechanisms. These mechanisms may present an extra advantage for the overall bacterial population as once earlier complement molecules such as C1q, C3 or MASPs have bound to the bacterial surface, they will not dissociate and deplete available complement components. Accordingly, terminal pathway resistance will allow the initial steps of the complement cascade to proceed but prevent MAC insertion, which may limit the overall complement response.

One of the most common terminal pathway resistance mechanisms in bacteria is through binding the protein Vn, which will bind C5b-7 or C5b-8 complexes and prevent them from being inserted into the outer membrane [28,32]. *H. influenzae* uses Protein E and Protein F to bind Vn [76]. *N. meningitidis* also possess multiple Vn binding proteins, such as Opc, Msf, and Hsf [77,78]. Both bacterial species do not possess many resistance mechanisms towards the earlier stages of the complement cascade, suggesting that there may be unknown advantages to inhibiting the later stages of complement. This may be a possible explanation as to why some bacteria appear to activate complement against themselves, only to stop the response in its latter stages.

Similar to binding Vn, Ogata and Levine [79] determined that *E. coli* uses the TraT protein to protect against complement-mediated killing. TraT is canonically known for its role in preventing the stable formation of mating pairs of cells carrying closely-related plasmids during conjugation [80]. It is thought that TraT prevents either the assembly or insertion of the MAC into the outer membrane. Hong et al. [81] determined that TraT inhibits the terminal pathway by binding C6 and prevents the formation of the C5b-6 complex and thus subsequent steps in MAC formation. It was also found that TraT is unable to inhibit the terminal pathway once C5b-6 complexes have been formed. Due to these findings of TraT imparting complement resistance, several Gram-negative bacterial species were screened for TraT and then tested for serum resistance. TraT was present in *Salmonella, Shigella,* and *Klebsiella* clinical isolates, but not all isolates were complement-resistant [82]. Conversely, Bitter-Suermann et al. [83] found that 51–56% of clinical *E. coli* strains expressed TraT on the outer membrane. They did not study the role of TraT in complement-resistance for these strains; nevertheless, these data suggest that TraT expression provides some advantage to *E. coli* clinical isolates. Further work to characterize TraT in complement resistance of *E. coli* clinical isolates will determine if its expression across strains and throughout infection would allow it to serve as a potential therapeutic target.

As an alternative to expressing outer membrane proteins to bind host-produced complement regulator proteins, *E. coli* can recruit such proteins directly into their outer membrane. For example, CD59 is an integral-membrane complement regulator protein that is typically bound to Eukaryotic cell membranes, protecting them from the terminal pathway of complement [28,32]. Rautemaa et al. [84] showed that *E. coli* could acquire CD59 to their outer membrane to provide protection against the complement system. It was found that even after several wash steps, CD59 had not been removed from the bacterial surface, and it was concluded that CD59 had integrated into the *E. coli* outer membrane. The authors hypothesized that the lipid A portion of *E. coli* LPS could interact with the glycosylphosphatidylinositol anchor of CD59, permanently tethering it to the bacterial surface. It is interesting that this complement-resistance mechanism has yet to be identified in other bacterial species considering the lipid A portion of the LPS is so well-conserved across species. If this mechanism is identified across multiple Gram-negative species, it could have the potential to be a broad-spectrum antibiotic target.

Conversely, some bacteria appear to have acquired Eukaryotic genes through HGT that allow them to resist the effects of complement. *Borrelia burgdorferi* is the causative agent of Lyme disease, and although not recognized as being highly antibiotic resistant, this species is able to form persister cells in the late stages of infection, rendering them extremely difficult to treat therapeutically [85]. *B. burgdorferi* expresses their own CD59-like protein and exports it to their outer membrane to protect themselves against complement [86]. This is the only documented case where a bacterium produces a complement regulator type protein that mimics the activity of complement regulators produced by the human host [86,87].

## 5. Mechanisms of Resistance

### 5.1. Physical Hindrance

Previously, most research focused on non-protein outer membrane structures such as LPS and capsule as complement resistance mechanisms. The O-antigen portion of LPS is largely responsible for protection against complement in Gram-negative bacteria as it prevents complement components from binding to the outer membrane [84]. This is due to steric hindrance impeding the complement components from attaching to their target molecule. Generally speaking, the longer the LPS O-antigen that the bacterium produces, the more complement protection the bacterium will likely possess. LPS side chains also prevent the MAC from inserting itself into the outer membrane, protecting the bacterium from the terminal pathway [84].

Interestingly, the molecular structure of the LPS does activate the alternative pathway in some bacteria. In order to protect themselves from complement, these bacteria may express a wide variety of protein-based resistance factors. However, some alter the structure of the LPS to recruit host-produced complement-regulator proteins. The most well-studied example of this is *N. meningococci* adding sialic acid to the LPS, which was characterized by Estabrook et al. [65]. In this case, the negative charge provided by the sialic acid recruits FH to the outer membrane. FH can then act as a cofactor to Factor I to inactivate C3b. The authors also determined that the degree of sialyation was positively correlated with complement-resistance. In summary, LPS presents a narrow-spectrum therapeutic target, and it is currently being researched in relation to antibacterial development [88].

Although the LPS has been well-characterized for many bacterial species, it is largely unknown how the asymmetry of the outer membrane is created. The Mla pathway was discovered in 2009 by Malinverni and Silhavy [60]. They identified that this pathway uses an ABC transport system to maintain the asymmetry of the outer membrane, with the LPS in the outer leaflet and phospholipids on the inner leaflet. Without this asymmetric structure, the outer membrane cannot function properly and therefore cannot provide sufficient protection against the complement system and other host stressors. The Mla pathway is not well-characterized in most species, but Mla genes have been identified as being essential to a wide variety of bacterial pathogens’ survival in the presence of complement. More specifically, VacJ mutants have been shown to play a critical role in complement-resistance for *H. influenzae*, *P. aeruginosa,* and *A. baumannii* [89,90,91]. VacJ corresponds to the *mlaA* gene in *E. coli*, where the MlaA lipoprotein is localized to the inner leaflet of the outer membrane [60]. MlaA is responsible for mediating the transport of phospholipids that have been removed from the outer leaflet of the outer membrane [60]. Thus, when the function of MlaA is destroyed, phospholipids are permitted into the outer leaflet of the outer membrane, and the outer membrane becomes sensitive to complement. Additionally, due to the reduction in the amount of LPS, it is likely that MAC can be inserted into the outer membrane more easily.

Because the Mla pathway is found in all Gram-negative bacteria, it has the potential to be a good therapeutic target for the development of broad-spectrum antibiotics. Drugs inhibiting the proper function of the Mla pathway would be targeting both a virulence factor and an important ancestral biological function, as seen before with moonlighting proteins. Therefore, drugs directed toward these targets would have similar advantages and disadvantages. However, drugs targeting the Mla pathway would face the additional challenge of having to cross the Gram-negative cell envelope to reach their target, where bacteria have a number of mechanisms to defend against such compounds entering the cell. This is in stark contrast to the ease with which MAbs could be designed toward surface exposed or secreted proteins, as it is not required that these drugs cross the cell wall. Therefore, the only likely resistance mechanism that could arise against the MAbs would be target alteration.

### 5.2. Complement Inhibitory Drugs

Many biotechnology companies are beginning to investigate monoclonal antibodies (MAbs) as a potential therapeutic option for novel antibacterials. To date, three MAbs have been approved by the US Food and Drug Administration targeting MDR

Gram-positive and Gram-negative bacteria [92]. Additionally, many MAbs are in pre-clinical or clinical trials targeting a variety of surface structures, including LPS which is a complement-resistance factor [92,93,94]. MAbs will bind to their designated target with high specificity and activate the complement response against the bacterium, which in turn will activate more sophisticated innate and adaptive immune responses [25,95]. The binding of a MAb to its target will also prevent the targeted protein from carrying out its normal function; in this case, the target will no longer provide complement-resistance to the bacterium. Targeting surface proteins with MAbs presents a variety of advantages. Most importantly, MAbs targeting surface proteins can directly kill the bacterium because they initiate the complement response against it. Additionally, MAbs that bind to complement resistance surface proteins render the pathogen more susceptible to the complement response.

MAbs targeting virulence factors such as complement resistance present a wide variety of advantages when compared to more traditional antibiotics targeting essential life processes. For example, because MAbs are highly specific to their target, they will not have additional effects on the natural microbiome of the host [95]. Recent findings in the field of the human microbiome have concluded that antibiotic treatment can drastically alter the composition of the microbiome once the patient has been recolonized [96,97]. Dysbiosis in the microbiome early in life has been linked to diseases and disorders such as asthma [98]. Due to a paucity of information in this field, it has become important to maintain a healthy microbiome, which could be facilitated via highly specific antibacterial therapies. Since we propose the use of MAbs targeting complement-resistance factors of pathogens, it is worth noting that very little research has been performed in the field of complement resistance of commensal microorganisms. It can be assumed that these bacteria must possess at least some form of resistance mechanisms against complement to survive; however, none have been specifically characterized to date other than LPS of Gram-negative bacteria. Therefore, we must proceed cautiously when claiming that MAbs directed towards pathogenic complement-resistance mechanisms have little to no effect on the microbiome.

Although MAbs directed at complement-resistance factors of pathogenic bacteria present many advantages, they also present challenges in terms of their development and use. Because of the high specificity of these drugs, rapid diagnostic tests capable of identifying the species of bacteria must be developed in parallel to these specific therapies [19,20,99]. Enzyme-linked immunoabsorbent assays (ELISA) could be used as a preliminary test to specifically detect the antigen to be targeted. This would be simple as the MAb designed for treatment could be used as a primary antibody to bind to the isolated bacteria, and detection could be performed with a labeled secondary antibody. Unfortunately, ELISAs are rather time-consuming, with results taking a minimum of 24 h to produce. Additionally, in order to produce the MAb, the protein to be bound by the MAb must be characterized at the molecular level [100,101]. That being said, it must be known that the clinical isolate to be treated expresses the virulence factor, which we are targeting throughout the entire infection [20,99,101]. Because the complement system is always active, it is likely that a pathogen will express its resistance factors throughout the entirety of the infection, but this has yet to be confirmed experimentally.

There are, however, some circumstances that may allow a pathogen to stop expressing complement-resistance factors. Biofilm formation has been an ongoing issue in antibiotic treatment and could potentially be a problem when using MAbs against surface-exposed complement-resistance proteins. Complement effectors and other host defense mechanisms cannot access the bacterial surface of all cells within a biofilm, rendering those cells more difficult to eradicate [102]. Because biofilms protect a large portion of bacteria from complement, it would be beneficial for bacteria to stop expressing complement-resistance factors and direct the energy and resources to different physiological processes involved in causing infection. However, the outside layer of the biofilm will always be exposed to complement, and it can be assumed these bacteria are expressing complement resistance factors. This leads to the theory that MAbs designed against complement-resistance factors could potentially be effective in treating biofilms. Initially, the MAb will be able to recognize its target on the outer layer of biofilm bacteria and initiate a complement attack against them, leading to cell lysis. Once this outside layer of cells has been removed, the inside layer will be exposed, which is assumed to be susceptible to complement. Therefore, at this point, the MAb is no longer necessary, and the complement cascade, as well as other immune responses, will be initiated against the inner layers of bacteria, in turn destroying the biofilm.

### 5.3. Secreted Complement Inhibitors

Some pathogens also use secreted proteases to inactivate complement components. *P. aeruginosa* uses the AprA metalloprotease to cleave C2, C1s, C3, and C4, providing protection against the classical and lectin pathways of complement [103]. Additionally, AprA is capable of resisting the terminal pathway by blocking the cleavage of C5 into C5a and C5b, which serves as the molecular scaffold for MAC assembly [103]. *P. aeruginosa* also possesses the secreted proteases elastase and alkaline protease, PaE and PaAP, respectively, which are capable of cleaving complement components C1q and C3 [81]. Similarly, *A. baumannii* has the PKF serine protease, which was found to inactivate human serum [104]. It was determined that PKF was cleaving various complement components, and it was hypothesized that it could be degrading properdin, which is necessary for the complement cascade as it stabilizes C3 and acts as a scaffold for the assembly of the C3bBb C3 convertase of the alternative pathway [104]. Secreted proteases appear to be an effective way to provide complement resistance to a bacterial population as not every bacterium must express the protease to be protected.

MAbs can also be used to target secreted proteases but present different advantages and disadvantages than MAbs targeting outer membrane proteins. To begin, MAbs targeting secreted proteins are easier to design to ensure strong binding [101]. In contrast, it is challenging to design MAbs against membrane proteins that bind to their target with high avidity. Although MAbs targeting secreted proteins are easier to design, they do not directly kill the pathogen but rather act to decrease its virulence, making it easier for the immune system to clear the infection [101]. Because of this, we predict MAbs would be most useful when delivered in combination with traditional antibiotics targeting essential processes.

## 6. Conclusions

Herein we discuss a wide variety of complement-resistance mechanisms utilized by Gram-negative bacterial pathogens that have the potential to become novel antibiotic targets. There are diverse options for which how antibiotics could target these mechanisms, including sequence-specific CRISPR-Cas drugs, MAbs targeting either surface-attached or secreted complement-resistance mechanisms, or more traditional small-molecule antibiotics capable of crossing the cell envelope to reach their target. Regardless of the type of drug developed, all the proposed antibacterials have a novel mechanism of action targeting immune evasion factors. These drugs have a decreased likelihood of resistance developing against them and could be suited for long-term use. We must continue to explore new options for antibacterial targets that exhibit similar properties to those discussed in this review if we wish to get out of the resistance era and avoid another devastating global pandemic.

## Figures and Tables

**Table 1:** Summary of complement-regulator proteins.

Complement Inhibitor	Type	Pathway It Inhibits	Ligand It Inhibits
Factor H (FH)	Fluid phase	Alternative	C3b
Factor H-Like 1 (FHL-1)	Fluid phase	Alternative	C3b
Factor H-Related 1 (FHR-1)	Fluid phase	Alternative	C3b and C5 convertase
		Terminal	C5 MAC scaffold
Plasminogen (Plg)	Fluid phase	All	C3 and C5 convertase
		Alternative	C3b
C4-Binding Protein (C4BP)	Fluid phase	Classical	C4bC2a C3 convertase
		Lectin	C4b
		Alternative	C3b
C1 Inhibitor (C1INH)	Fluid phase	Classical	C1s and C1r
		Lectin	MASP-1 and MASP-2
		Alternative	C3b
Victronectin (Vn)	Fluid phase	Terminal	Binds C5b-7, allows full assembly of MAC but prevents insertion
			OR
			Bind C5b-8 preventing MAC formation
Clusterin (Cn)	Fluid phase	Terminal	Same as Vn and C5b-9
CD59/Protectin	Surface attached	Terminal	Prevent C9 association with C5b-8

**Table 2:** Summary of complement-resistance mechanisms used by Gram-negative bacteria.

Bacteria	Complement-Resistance Mechanism	Complement Pathway Inhibited	Mechanism of Action
*E. coli*	StcE	All	Binds host C1INH
	Mla pathway	All or Terminal	Establishes and maintains membrane stability
	Host-produced CD59	Terminal	Acquired to OM
	TraT	Terminal	Inhibits host C6 preventing MAC formation
	Curli	Classical	↓ host C1q deposition
*P. aeruginosa*	Lpd	Alternative and Terminal	Binds host FH, FHL-1, FHR-1
	Tuf	Alternative	Binds host FH, FHL-1, plasminogen
	AprA	All	Cleaves host C2, C1s, C3, C4, C5
	Porin D	Terminal	Binds host Vn
	Mla Pathway	All OR Terminal	Establishes and maintains membrane stability
*H. influenzae* Type B	Protein E (PE)	Terminal	Binds host Vn and Plasminogen
	Protein F (PF)	Terminal	Binds host Vn
	Mla Pathway	All or Terminal	Establishes and maintains membrane stability
*A. baumanii*	OmpA	Alternative	Binds host FH
	CipA	Terminal	Binds host Plasminogen
	Tuf	Terminal	Binds host Plasminogen
	PKF	All	Potentially degrading host Properdin, destabilizing C3
	PBP-7/8	All or Terminal	Cell wall biogenesis
	Mla Pathway	All or Terminal	Establishes and maintains membrane stability
*B. pertussis*	BvgAS	All	Binds host C1INH
	FHA	All	Binds host C4BP
	BrkA	Classical and Alternative	↓ host C4 and C3 deposition
	BapC	Unknown	Unknown
*N. meningitidis*	NspA	Alternative	Binds host FH
	Opc	Terminal	Binds host Vn
	Msf	Terminal	Binds host Vn
	Hsf	Terminal	Binds host Vn
	PPX	All and Terminal	↓ host C3 and MAC deposition
	Pilin	All	Causes clumping of cells

## Data Availability

Not applicable.
